# Community Emergency Care Use by Veterans in an Era of Expanding Choice

**DOI:** 10.1001/jamanetworkopen.2024.1626

**Published:** 2024-03-08

**Authors:** Anita A. Vashi, Tracy Urech, Siqi Wu, Linda D. Tran

**Affiliations:** 1 Center for Innovation to Implementation, Veterans Affairs Palo Alto Health Care System, Menlo Park, California; 2 Department of Emergency Medicine, University of California, San Francisco; 3 Department of Emergency Medicine (Affiliated), Stanford University, Stanford, California; 4 Stanford Primary Care and Population Health, Stanford University, Stanford, California; 5 Health Economics Resource Center, Veterans Affairs Palo Alto Health Care System, Palo Alto, California; 6 Surgery Policy Improvement and Education Center, Stanford Medicine, Stanford University, Stanford, California

## Abstract

**Importance:**

Recently passed legislation aimed at improving access to care has considerably expanded options for veterans to receive emergency care in community, or non–Veterans Affairs (VA) settings. However, national trends in community emergency department (ED) use by veterans are unknown.

**Objective:**

To examine national, temporal trends in the frequencies and types of ED visits provided in community settings and explore the association between facilities’ purchase of community care with facility and regional characteristics.

**Design, Setting, and Participants:**

Retrospective, observational cross-sectional study of ED visits over fiscal years (FY) 2016 to 2022. VA and community ED encounter data were obtained from the VA Corporate Data Warehouse and the Office of Integrated Veteran Care. Participants were veterans receiving ED care at VA facilities or paid for by the VA in the community. Data were analyzed from June to September 2023.

**Main Outcomes and Measures:**

The primary outcome measures included community ED visit volume, disposition, and payments over time. Also, the most common and costly ED visits were assessed. Negative binomial regression analysis examined associations between facility and regional characteristics and the rate of ED visits purchased in community settings relative to all ED visits.

**Results:**

There were 19 787 056 ED visits, predominantly at VA facilities (14 532 261 visits [73.4%]), made by 3 972 503 unique veterans from FY 2016 to 2022. The majority of ED users were male (3 576 120 individuals [90.0%]), and the median (IQR) age was 63 (48-73) years. The proportion of community ED visits increased in absolute terms from 18% in FY 2016 to 37% in FY 2022. Total community ED payments, adjusted to 2021 dollars, were $1.18 billion in FY 2016 and over $6.14 billion in FY 2022. The most common reasons for ED visits in the community were for nonspecific chest pain (305 082 visits [6%]), abdominal pain (174 836 visits [3%]), and septicemia (149 968 visits [3%]). The average proportion of ED visits purchased by a VA facility increased from 14% in FY 2016 to 32% by FY 2022. In multivariable analyses, facilities with greater ED volume and low-complexity facilities had higher expected rates of community emergency care than lower volume and high-complexity facilities, respectively.

**Conclusions and Relevance:**

As veterans increasingly use community EDs for acute, unscheduled needs, attention to factors associated with veterans’ use of acute care services in different settings are important to identify access barriers and to ensure veterans’ health care needs are met.

## Key Points

**Question:**

What are the national, temporal trends in the frequencies and types of emergency department (ED) visits provided in community settings, and what characteristics are associated with facilities’ purchase of community ED care?

**Findings:**

In this cross-sectional study of more than 19 million ED visits, the proportion of community ED visits increased in absolute terms from 18% in fiscal year 2016 to 37% in fiscal year 2022. Low-complexity facilities were more likely to purchase community emergency care than their high-complexity counterparts.

**Meaning:**

These findings suggest that with a pronounced shift toward non–Veterans Affairs ED care, policies are needed to minimize fragmented patient experiences across care settings and navigate the complexities of providing and purchasing community emergency care.

## Teaser

This cross-sectional study evaluates the national and temporal trends in the frequencies and types of emergency department visits provided to veterans in community settings and the association between Veterans Affairs facilities’ purchase of community care with facility and regional characteristics.

## Introduction

The Veterans Affairs (VA) Health Care System serves as the largest publicly financed integrated national health care system in the US, treating over 9 million patients through an extensive network of over 1300 facilities.^1^ To serve the acute care needs of its population, the VA has developed an emergency care system that provides acute, unscheduled care across 110 emergency departments (EDs).^2^

Accessing emergency care is challenging for veterans who rely on the VA for health care, particularly in rural and underserved areas, in part because of the limited geographic reach of the VA emergency care system. One solution to mitigate this access challenge has been to purchase care in the private, or non-VA, sector, thereby allowing eligible veterans to use their benefits outside the VA for treatment services delivered by practitioners in closer proximity.

Recent legislation, particularly the VA Maintaining Internal Systems and Strengthening Integrated Outside Networks (MISSION) Act of 2018, significantly expanded opportunities for veterans to receive care in non-VA settings (hereafter referred to as community care or VA purchased care), thereby transforming the VA from a traditional source of health care services to both an integrated source and purchaser of health care.^3^ By 2021, almost one-third of veterans had received some type of community care. Implementation of the MISSION Act and concomitant changes in emergency care payment authorities, notification processes, and reimbursement rates have also simplified the process of approving and paying for community emergency care.^4^ Consequently, the VA has witnessed an unprecedented surge in demand for community-based emergency care. Emergency care has emerged as the primary contributor to VA community care spending, with community ED visit expenditures rising by 46% since 2020.^2^

With its dual role as a source and purchaser, the VA is faced with new challenges in delivering and coordinating acute care services across multiple settings. For veterans, use of both VA and community ED care may lead to duplicative and/or inefficient care, medical errors, adverse clinical outcomes, and less favorable patient experiences.^5^ The unprecedented expansion of community care may pose sustainability challenges for VA policymakers, potentially placing a strain on the fiscal solvency of the VA’s community care program. Since there have been no prior efforts to assess temporal trends in the rate of community ED visits, hospital admission rates, and ED payments, we used nationally representative data to examine temporal trends in community ED visits among veterans between 2016 and 2022 and explored the association between facilities’ purchase of emergency care and facility and regional factors. Findings from this study will provide the VA with preliminary data that can help facilitate decision-making and policies related to the complexities of providing and purchasing emergency care.

## Methods

### Study Design and Population

This was a retrospective study using VA outpatient encounter data and community care data obtained from the VA Corporate Data Warehouse (CDW), a national repository comprising data from several VA clinical and administrative systems.^6^ The overall study sample included all ED visits provided or purchased by the VA between October 1, 2015, and September 30, 2022, reflecting VA fiscal years (FY) 2016 to 2022, by veterans who were aged 18 years or older. Each visit was considered an independent observation. The Stanford University institutional review board determined that the evaluation did not meet the federal definition of research and did not require approval or informed consent. This study followed the Strengthening the Reporting of Observational Studies in Epidemiology (STROBE) reporting guideline.

### ED Encounter Variables

ED visit dates, diagnosis codes, and visit disposition (treated and released, admitted, or died) for VA and community care ED visits were derived from the CDW and from claims provided by the VA’s Office of Integrated Veteran Care, respectively. VA ED visits were identified using stop codes used by the VA to classify clinical groups and specialties responsible for the care provided. The Office of Integrated Veteran Care identified community ED visits through procedure codes and/or revenue codes reported in community care claims. Using the primary diagnosis, diagnosis codes were classified into clinically meaningful and mutually exclusive categories via the Agency for Healthcare Research and Quality Clinical Classifications Software Refined (CCSR).^7^ Total payments for community care ED visits were obtained from the VA’s Office of Integrated Veteran Care Community Care ED claims and calculated based on the program office’s guidance. Payments for community ED visits reflect both the ED care and subsequent hospitalization (for ED admissions only). The VA facility accountable for payment of a community emergency encounter is recorded in the claims and typically corresponds to the VA facility nearest to the location where the care was administered, or the VA facility associated with the patient.

For each encounter, we used demographic and clinical information from the CDW to describe the veteran’s age, race, sex, service-connected disability rating, housing status, rurality of residential location, and Elixhauser Comorbidity Index.^8^ We included race given racial disparities in health care. For more detail regarding variable specification, please see eMethods in Supplement 1.

### Facility-Level Variables

Available VA facility-level information included presence of an ED, teaching status, facility complexity, ED volume, rural status of facility location, and US Census division. Teaching status was defined using 2018 American Hospital Association data. Facility complexity describes the level of services provided at a VA facility—categorized as 1a, 1b, 1c, 2, or 3—with level 1a being the most complex and level 3 being the least complex (see eMethods in Supplement 1 for details). ED volume was calculated for each fiscal year.

### Statistical Analysis

We examined ED visit volume and disposition by setting of care (VA vs community) and over time. Next, we summarized the characteristics of all VA patients who received emergency care in the community by fiscal year. We then described trends in community ED payments (adjusted to 2021 dollars) and identified the most common and costly ED diagnosis categories for community ED visits. To explore variation in the types of conditions purchased in the community, we calculated the proportions of community ED visits vs VA ED visits for each of the CCSR categories.

In facility-level analyses, we first examined associations between facility or regional characteristics and the proportion of ED visits purchased in the community relative to all ED visits per fiscal year using Wilcoxon signed-rank or Kruskal-Wallis tests. We then used negative binomial regression to model rates of community ED visits on facility characteristics and US region. Cluster-robust standard errors were estimated to address intergroup association within facilities. Analyses were limited to facilities with an active ED during the year (750 facility-years; 110 unique facilities). Negative binomial regression results are reported as incidence rate ratios (IRRs) with 95% CIs. A 2-sided *P* < .05 was considered statistically significant. All analyses were performed using Stata version 18.0 (StataCorp). Data were analyzed from June to September 2023.

## Results

There were 19 787 056 ED visits, predominantly at VA facilities (14 532 261 visits [73.4%]), made by 3 972 503 unique veterans from FYs 2016 to 2022. The majority of ED users were male (3 576 120 individuals [90.0%]), and the median (IQR) age was 63 (48-73) years. The annual number of community ED visits increased 154%, from 465 253 in FY 2016 to 1 180 106 in FY 2022, while the number of unique users of community emergency care increased by 134%, from 304 512 to 712 111 (Figure). The proportion of all ED visits that occurred in the community progressively increased from 18% (465 253 visits) to 37% (1 180 106 visits).

**Figure.**
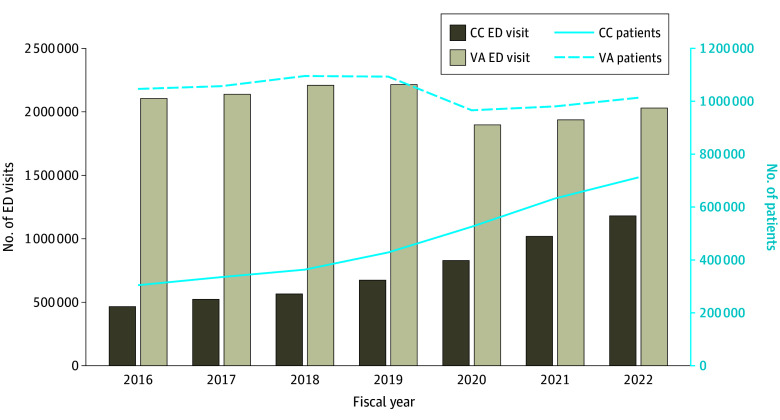
Number of Community Care (CC) and Veterans Affairs (VA) Emergency Department (ED) Visits and Unique Patients, Fiscal Years 2016 to 2022

Table 1 summarizes encounter-level patient characteristics of community ED visits. Patient median (IQR) age for all community ED visits during the study period was 64 (51-73) years. Over time, patients seen in community EDs shifted toward an older population. Patients aged 76 and older represented only 41 580 community ED visits (9%) in FY 2016 and increased to 276 798 (23%) by FY 2022. This trend coincides with a decrease in the proportion of patients in the low-income priority group, a decrease in unhoused patients, and an increase in patients with fewer comorbid Elixhauser conditions. Patient sex, race, and ethnicity were stable during the study period (Table 1; eTable 1 in Supplement 1 for characteristics of patients).

**Table 1.** Patient Characteristics of Community Emergency Department (ED) Visits Purchased by Veterans Affairs (VA), Fiscal Years 2016 to 2022^a^

Characteristic^b^	Patients, No. (%)							
	2016 (n = 304 512)	2017 (n = 335 095)	2018 (n = 363 425)	2019 (n = 428 312)	2020 (n = 525 499)	2021 (n = 632 481)	2022 (n = 712 111)	Overall (N = 1 941 414)
Total ED visits, No.	465 253	522 280	565 014	674 639	828 362	1 019 141	1 180 106	5 254 795
Age, y, mean (SD)	58.1 (15.4)	58.6 (15.5)	59 (15.7)	59.8 (16)	62.1 (16.2)	62.8 (16.3)	63.6 (16.5)	61.3 (16.2)
Age group								
18-35	57 233 (12.3)	63 023 (12.1)	65 680 (11.6)	73 769 (10.9)	74 361 (9.0)	85 476 (8.4)	94 228 (8.0)	513 770 (9.8)
36-50	71 915 (15.5)	81 306 (15.6)	89 988 (15.9)	108 100 (16.0)	119 321 (14.4)	147 625 (14.5)	165 943 (14.1)	784 198 (14.9)
51-64	153 096 (32.9)	161 908 (31.0)	167 493 (29.6)	188 347 (27.9)	208 338 (25.2)	239 874 (23.5)	261 721 (22.2)	1 380 777 (26.3)
65-75	141 266 (30.4)	166 739 (31.9)	183 631 (32.5)	220 350 (32.7)	278 399 (33.6)	339 672 (33.3)	380 472 (32.2)	1 710 529 (32.6)
76-100	41 580 (8.9)	49 154 (9.4)	58 018 (10.3)	83 781 (12.4)	147 514 (17.8)	205 803 (20.2)	276 798 (23.5)	862 648 (16.4)
Sex								
Male	414 937 (89.2)	465 103 (89.1)	500 366 (88.6)	598 308 (88.7)	744 698 (89.9)	915 707 (89.9)	1 055 658 (89.5)	4 694 777 (89.3)
Female	50 287 (10.8)	57 159 (10.9)	64 621 (11.4)	76 293 (11.3)	83 630 (10.1)	103 360 (10.1)	124 399 (10.5)	559 749 (10.7)
Race								
AIAN	5053 (1.1)	5862 (1.1)	6238 (1.1)	7444 (1.1)	8858 (1.1)	10 468 (1.0)	11 882 (1.0)	55 805 (1.1)
Asian	2414 (0.5)	3060 (0.6)	3246 (0.6)	3965 (0.6)	4301 (0.5)	5033 (0.5)	6144 (0.5)	28 163 (0.5)
Black or African American	76 755 (16.5)	88 728 (17.0)	98 526 (17.4)	116 895 (17.3)	130 605 (15.8)	159 504 (15.7)	183 026 (15.5)	854 039 (16.3)
NHOPI	4636 (1.0)	5592 (1.1)	5786 (1.0)	6806 (1.0)	7539 (0.9)	9159 (0.9)	10 115 (0.9)	49 633 (0.9)
White	347 873 (74.8)	387 628 (74.2)	417 479 (73.9)	498 748 (73.9)	626 227 (75.6)	769 959 (75.6)	890 532 (75.5)	3 938 446 (75.0)
Unknown	28 522 (6.1)	31 410 (6.0)	33 739 (6.0)	40 781 (6.0)	50 832 (6.1)	65 018 (6.4)	78 407 (6.6)	328 709 (6.3)
Ethnicity								
Hispanic or Latino	27 195 (5.9)	31 115 (6.0)	34 690 (6.1)	41 896 (6.2)	45 140 (5.5)	54 863 (5.4)	61 656 (5.2)	296 555 (5.6)
Not Hispanic or Latino	427 161 (91.8)	478 793 (91.7)	516 900 (91.5)	615 873 (91.3)	759 980 (91.7)	927 845 (91.0)	1 054 185 (89.3)	4 780 737 (91.0)
Unknown	10 897 (2.3)	12 372 (2.4)	13 424 (2.4)	16 870 (2.5)	23 242 (2.8)	36 433 (3.6)	64 265 (5.5)	177 503 (3.4)
Unhoused	61 086 (13.1)	66 852 (12.8)	68 260 (12.1)	74 911 (11.1)	81 244 (9.8)	89 621 (8.8)	97 305 (8.3)	539 279 (10.3)
Rurality								
Urban	275 120 (59.1)	307 874 (59.0)	332 100 (58.8)	392 593 (58.2)	466 875 (56.4)	570 538 (56.0)	646 146 (54.8)	299 1246 (56.9)
Rural	189 053 (40.6)	212 080 (40.6)	231 211 (40.9)	279 968 (41.5)	359 616 (43.4)	446 826 (43.9)	521 796 (44.2)	2 240 550 (42.6)
Insular	325 (0.1)	753 (0.1)	724 (0.1)	1036 (0.2)	858 (0.1)	878 (0.1)	969 (0.1)	5543 (0.1)
VA priority group^b^^,^^c^								
Highly disabled	252 994 (54.4)	291 798 (55.9)	327 345 (57.9)	389 008 (57.7)	438 234 (52.9)	535 616 (52.6)	642 626 (54.5)	2 877 621 (54.8)
Low/moderate disability	71 654 (15.4)	79 017 (15.1)	83 536 (14.8)	102 511 (15.2)	139 093 (16.8)	176 494 (17.3)	203 172 (17.2)	855 477 (16.3)
Low income	110 723 (23.8)	117 284 (22.5)	117 873 (20.9)	137 262 (20.4)	176 397 (21.3)	203 770 (20.0)	217 769 (18.5)	1 081 078 (20.6)
Nondisabled	29 587 (6.4)	33 861 (6.5)	35 974 (6.4)	45 367 (6.7)	74 067 (8.9)	102 745 (10.1)	116 188 (9.9)	437 789 (8.3)
VA SC disability rating								
No SC disability	156 971 (33.7)	167 715 (32.1)	171 345 (30.3)	202 326 (30.0)	281 773 (34.0)	345 202 (33.9)	376 977 (31.9)	1 702 309 (32.4)
SC 0%-49%	76 073 (16.4)	83 816 (16.1)	88 059 (15.6)	107 775 (16.0)	142 902 (17.3)	179 371 (17.6)	205 258 (17.4)	883 254 (16.8)
SC 50%-99%	137 159 (29.5)	158 251 (30.3)	176 089 (31.2)	209 753 (31.1)	237 053 (28.6)	289 875 (28.4)	339 932 (28.8)	1 548 112 (29.5)
SC 100%	93 978 (20.2)	11 1286 (21.3)	128 071 (22.7)	152 697 (22.6)	163 252 (19.7)	200 669 (19.7)	252 118 (21.4)	1 102 071 (21.0)
Elixhauser comorbidity score, mean (SD)	4.3 (2.8)	4.4 (3.0)	4.6 (3.2)	4.7 (3.3)	4.4 (3.2)	4.3 (3.1)	3.9 (2.8)	4.3 (3.1)
Elixhauser conditions								
0-1	105 199 (22.6)	117 209 (22.4)	122 255 (21.6)	144 819 (21.5)	213 483 (25.8)	276 029 (27.1)	345 933 (29.3)	1 324 927 (25.2)
2-3	139 135 (29.9)	152 285 (29.2)	159 310 (28.2)	188 950 (28.0)	245 940 (29.7)	308 550 (30.3)	372 013 (31.5)	1 566 183 (29.8)
4-6	139 795 (30.1)	153 775 (29.4)	166 042 (29.4)	197 859 (29.3)	226 135 (27.3)	272 388 (26.7)	315 617 (26.7)	1 471 611 (28.0)
≥7	81 124 (17.4)	99 011 (19.0)	117 407 (20.8)	143 011 (21.2)	142 804 (17.2)	162 174 (15.9)	146 543 (12.4)	892 074 (17.0)

Abbreviations: AIAN, American Indian and Alaska Native; NHOPI, Native Hawaiian or Other Pacific Islander; SC, service-connected.

^a^ Table created using encounter-level data so that a patient with multiple visits contributes multiple times. For patient-level characteristics, please see eTable 1 in Supplement 1.

^b^ Values expressed as percentages unless otherwise indicated. Missing values for age, sex, rurality, VA priority group, and VA SC disability characteristics were less than 1.0% and are not reported in the table.

^c^ Highly disabled defined as VA Priority Groups 1 and 4. Low/moderate disability defined as VA Priority Groups 2, 3, and 6. Low income defined as VA Priority Group 5. Nondisabled defined as VA Priority Groups 7 and 8.

Community ED admission rates increased from 22% in FY2016 (101 576 admissions) to their highest rate of 26% in FY2021 (263 016 admissions), which coincided with steep increases in total community ED payments per year (eFigure in Supplement 1). Total community care ED payments, adjusted to 2021 dollars, were $1.18 billion in FY 2016. By FY 2022, the VA paid approximately $6.15 billion for community ED care. Median (IQR) ED payments per visit ($13 081 [$9170-$18 814] in FY 2022) increased 125% from FY 2016 to FY 2022 for ED admissions compared with 79% increase for ED treat-and-release visits ($752 [$381-$1 246] in FY 2022).

The most common reasons for community ED visits were for nonspecific chest pain (305 082 visits [6%]), abdominal pain (174 836 visits [3%]), and septicemia (149 968 visits [3%]), but varied by disposition (Table 2). While the most common reasons for community ED visits were stable over time, in FY 2021 and 2022, COVID-19 (5%) replaced abdominal pain as the second most common diagnosis category (eTable 2 in Supplement 1). The costliest conditions treated in community EDs during the study period were septicemia, acute myocardial infarction, and COVID-19 (eTable 3 in Supplement 1). The conditions (>1000 ED visits) with the highest median (IQR) ED charges in 2022 were fracture of the neck of the femur ($18 882 [$14 066-$24 822]), conduction disorders ($19 695 [$2 813-$25 149]), and septicemia ($16 646 [$13 361-$21 952]).

**Table 2.** Most Common Conditions Treated in Community Emergency Departments by Visit Disposition, Fiscal Years 2016 to 2022

CCSR condition category	Rank	Patients, No. (%)
Treat and release (n = 4 003 658)		
Nonspecific chest pain	1	293 200 (7.3)
Abdominal pain	2	172 168 (4.3)
Musculoskeletal pain, not low back pain	3	133 932 (3.4)
Superficial injury; contusion	4	122 504 (3.1)
Sprains and strains	5	105 207 (2.6)
Respiratory signs and symptoms	6	104 780 (2.6)
Open wounds to limbs	7	98 460 (2.5)
Skin and subcutaneous tissue infections	8	91 625 (2.3)
Chronic obstructive pulmonary disease and bronchiectasis	9	84 100 (2.1)
Spondylopathies/spondyloarthropathy	10	80 370 (2.0)
Admitted (n = 1 240 671)		
Septicemia	1	137 718 (11.1)
Heart failure	2	78 942 (6.4)
Acute myocardial infarction	3	51 862 (4.2)
Cerebral infarction	4	40 376 (3.3)
COVID-19	5	39 196 (3.2)
Cardiac dysrhythmias	6	37 910 (3.1)
Chronic obstructive pulmonary disease and bronchiectasis	7	37 869 (3.1)
Pneumonia	8	36 094 (2.9)
Diabetes with complication	9	33 465 (2.7)
Acute and unspecified kidney failure	10	33 292 (2.7)

Abbreviation: CCSR, Clinical Classification Software Refined.

There was variation in the types of conditions that presented to community care relative to the VA over the study period (Table 3). Among the 10 most common conditions seen overall (VA and community EDs), roughly one-third of nonspecific chest pain (305 082 cases [36%]) and chronic obstructive pulmonary disease–related ED visits (121 932 cases [33%]) occurred in the community, while only 59 910 low back pain cases (10%) and 5472 other specified encounters and counseling-related visits (2%) were seen in the community. The conditions with the greatest share of ED visits occurring in the community included pregnancy complications (13 547 [92%]), aspiration pneumonitis (10 288 [88%]), and cardiac arrest and ventricular fibrillation (15 053 [86%]) (Table 3).

**Table 3.** Distribution of Conditions Across Emergency Department (ED) Settings of Care, Fiscal Years 2016 to 2022

Rank	CCSR condition category	Total ED visits	Community, No. (%)	VA, No. (%)
Among most common reasons for ED visits (overall)				
1	Musculoskeletal pain, not low back pain	1 086 979	134 780 (12.4)	952 199 (87.6)
2	Nonspecific chest pain	839 157	305 082 (36.4)	534 075 (63.6)
3	Abdominal pain	731 271	174 836 (23.9)	556 435 (76.1)
4	Low back pain	601 937	59 910 (10.0)	542 027 (90.0)
5	Skin and subcutaneous tissue infections	563 815	111 446 (19.8)	452 369 (80.2)
6	Respiratory signs and symptoms	558 070	106 913 (19.2)	451 157 (80.8)
7	Other specified upper respiratory infections	380 511	44 236 (11.6)	336 275 (88.4)
8	Urinary tract infections	379 134	90 917 (24.0)	288 217 (76.0)
9	Chronic obstructive pulmonary disease and bronchiectasis	375 577	121 932 (32.5)	253 645 (67.5)
10	Other specified encounters and counseling	369 221	5472 (1.5)	363 749 (98.5)
Among conditions with highest proportion of visits occurring in Community EDs^a^				
1	Septicemia	198 161	149 968 (75.7)	48 193 (24.3)
2	Cerebral infarction	84 677	53 225 (62.9)	31 452 (37.1)
3	Traumatic brain injury; concussion	50 122	31 838 (63.5)	18 284 (36.5)
4	Fracture of the neck of the femur (hip)	29 169	21 185 (72.6)	7984 (27.4)
5	Poisoning by drugs	26 024	21 034 (80.8)	4990 (19.2)
6	Complication of cardiovascular device, implant or graft	24 074	15 004 (62.3)	9070 (37.7)
7	Cardiac arrest and ventricular fibrillation	17 560	15 053 (85.7)	2507 (14.3)
8	Acute hemorrhagic cerebrovascular disease	17 461	11 209 (64.2)	6265 (35.8)
9	Other specified complications in pregnancy	14 776	13 547 (91.7)	1229 (8.3)
10	Aspiration pneumonitis	11 689	10 288 (88.0)	1401 (12.0)

Abbreviations: CCSR, Clinical Classification Software Refined; ED, emergency department, VA, Veterans Affairs.

^a^ Limited to CCSR categories with greater than 10 000 community ED visits.

Among 110 facilities included in analyses, a mean (SD) of 5519 (4413) ED visits were purchased per fiscal year, ranging from 1% (79 visits) to 73% (12 779 visits) per year; the mean (SD) was 22% (12%), which increased from 14% (8%) in FY 2016 to 32% (13%) by FY 2022. Bivariate statistics are presented in eTable 4 in Supplement 1.

Negative binomial regression results are detailed in Table 4. The expected rate of ED visits purchased from the community was at least 2 times higher in recent years compared with 2016. Controlling for other factors in the model, the expected rate of community emergency care for medium (IRR, 1.53; 95% CI, 1.19-1.95; *P* = .001) and low (IRR, 1.51; 95% CI, 1.16-1.96; *P*  = .002) complexity facilities was 53% and 51% higher than high complexity facilities, respectively. Estimates translated to 613 (95% CI, 198-1029) to 5066 (95% CI, 1518-8614) additional community ED visits per fiscal year, depending on facility ED volume. Facilities with greater ED volume also had higher expected rates of community emergency care compared with facilities with 10 000 or fewer visits per fiscal year. While the rate of community ED visits was significantly associated with US Census division, estimates for teaching facility status and rural status were not statistically significant.

**Table 4.** Facility Factors Associated With the Rate of Emergency Department (ED) Visits Purchased in the Community

Characteristic	Incidence rate ratio (95% CI)	Robust standard error	*P* value
Fiscal year			
2016	NA	NA	NA
2017	NA	0.017	<.001
2018	NA	0.030	<.001
2019	NA	0.035	<.001
2020	NA	0.055	<.001
2021	NA	0.071	<.001
2022	NA	0.077	<.001
ED visit volume			
10 000 or fewer visits	1 [Reference]	NA	NA
10 001-20 000	1.28 (1.03-1.59)	0.140	.02
20 001-30 000	1.23 (0.98-1.56)	0.146	.08
≥30 001	1.60 (1.22-2.10)	0.220	.001
VA facility complexity			
1a: High complexity	1 [Reference]	NA	NA
1b: High complexity	0.91 (0.77-1.08)	0.080	.29
1c: High complexity	1.12 (0.93-1.36)	0.111	.24
2: Medium complexity	1.53 (1.19-1.95)	0.192	.001
3: Low complexity	1.51 (1.16-1.96)	0.203	.002
Teaching facility^a^	0.94 (0.80-1.10)	0.076	.43
US Census division			
East North Central	1 [Reference]	NA	NA
East South Central	1.50 (1.12-2.01)	0.224	.006
Middle Atlantic	0.69 (0.50-0.94)	0.111	.02
Mountain	1.17 (0.89-1.54)	0.162	.26
New England	1.19 (0.93-1.51)	0.146	.16
Pacific	1.18 (0.84-1.66)	0.205	.34
South Atlantic	0.99 (0.79-1.24)	0.114	.93
West North Central	1.10 (0.85-1.42)	0.144	.46
West South Central	1.27 (0.98-1.63)	0.162	.07
Rural facility^a^	1.22 (0.95-1.57)	0.157	.12
Constant (baseline incidence rate)	0.09 (0.06-0.12)	0.014	<.001

Abbreviations: NA, not applicable; VA, Veterans Affairs.

^a^ Reference groups for teaching and rurality were nonteaching facilities and facilities located in urban areas, respectively.

## Discussion

This study examined national trends in utilization of emergency care by veterans in community settings over the study time period as well as explored facility variation in how much emergency care is purchased in the community as a proportion of VA and community care use. Between FYs 2016 and 2022, the total annual visits to community EDs increased by 23%, corresponding to just over 1.1 million additional ED visits nationwide. This is despite a fairly stable population over the same period (eTable 5 in Supplement 1).

Our findings underscore a pivotal transformation in the acute care landscape for veterans, with a pronounced shift toward community-based emergency care, especially following the MISSION Act’s implementation in 2019. While this shift likely signifies an enhancement in access to care, it is essential to recognize its potential unintended repercussions. Notably, increased reliance on community emergency care can lead to concerns regarding health care outcomes and care coordination, potentially resulting in fragmented patient experiences across care settings.^9,10^ Moreover, the observed surge in community care ED visits may be partially attributed to demographic shifts, particularly the increasing proportion of elderly veterans, who may present a higher burden of illness. Nevertheless, the shift to community emergency care is noteworthy given prior research indicating that veterans have historically been hesitant to embrace community-based emergency care.^11^ The juxtaposition of these findings underscores the need for further investigation into the factors influencing veterans’ health care-seeking behaviors, as it is conceivable that as veterans grow more accustomed to seeking care in the community, community care ED utilization might continue to rise.

Due to the unpredictable nature of emergent care, emergency care is often authorized by the VA in certain circumstances when the VA is notified within 72 hours of a community ED visit. We found that the increase in community care ED utilization has resulted in an exponential increase in costs to the VA. One explanation for this surge in costs could be attributed to a phenomenon known as payer-shifting, a concept supported by prior research.^4^ MISSION Act related changes in payment and notifications, particularly reimbursing most community ED claims at 100% of Medicare rates, may have inadvertently created incentives leading to a transition in the primary payer for emergency care. This shift, from Medicare or other private payers to the VA, has critical implications but raises the question of whether it is primarily associated with patient choices or clinician practices, an issue warranting exploration in future research. Moreover, our findings underscore that a major reason for this cost escalation is closely tied to ED admissions. Although comparisons between the cost of VA-delivered and community care are limited, the VA has the ability to manage and standardize the care that it delivers directly, but it is not able to manage veterans’ care once they are in the care of community practitioners. Consequently, VA policy makers are actively considering repatriation strategies, which involve transferring veterans from community settings to VA facilities after initial stabilization and treatment (Care Optimization in the Emergency Department Integrated Project Team, personal communication, September 18, 2023, document). This approach carries several potential advantages, such as mitigating care fragmentation and theoretically decreasing costs by capitalizing on the VA’s comprehensive medical records and reducing redundant or unnecessary testing. However, these benefits come with inherent risks, including treatment delays, the possibility of incurring additional expenses related to the cost of transport and other required resources such as staffing. It is also not known to what extent VA facilities vary in their capacity to accept and treat (eg, bed availability, specialist availability) patients from the community. Additionally, the repatriation process itself introduces risks such as infection, airway complications, and cardiac arrest.^12,13^ We recommend that future research examine the cost-benefit analysis of repatriation strategies for veterans admitted to community hospitals.

Our study further highlights the significant consequences of the escalating utilization of community care EDs on the VA’s financial landscape. VA spending on community care has surged from $7.9 billion in 2014 to $18.5 billion in 2021.^14^ As community care costs have continued to mount, so too has the percentage of the VA budget allocated to this category. In 2014, community care accounted for approximately 12% of the VA’s financial resources; however, this allocation has grown to a substantial 33% of the VA medical care budget, sparking concerns among policymakers about the long-term sustainability of the community care program.^15^ Of particular relevance to our findings is the fact that emergency care now encompasses over one-third of the total community care expenditure. This observation underscores the urgency of addressing the underlying factors contributing to this budgetary surge and seeking solutions for a potentially unsustainable trend.

While the primary reasons for ED visits were fairly consistent over time, it is noteworthy that there existed a significant variation in the distribution of these visit types between community ED and VA settings. Notably, a substantial prevalence of pregnancy-related complications and trauma-related cases, such as hip fractures and organ injuries, were observed in community EDs. This observation likely stems from the fact that while VA EDs, although diverse in their capabilities, generally do not function as major trauma centers, serve a smaller population of female patients, and commonly refer obstetrical cases to community facilities.^16^ Additionally, a higher prevalence of conditions considered to be emergency care sensitive, such as cardiac arrest, hemorrhagic stroke, and sepsis, which necessitate timely access to emergency interventions for improved outcomes, might be influenced, at least in part, by proximity to community ED facilities. This suggests that the choice of care settings by veterans may be influenced by the specific nature of their medical needs. Understanding the factors associated with these patterns warrants more focused investigation in future research.

As expected, less complex facilities were more likely to purchase emergency care in the community than their more complex counterparts. This tendency likely mirrors the greater capacity of complex VA facilities to deliver a comprehensive array of timely acute care services, such as access to specialty consultants and advanced imaging resources.^17^ Since this was a preliminary, descriptive study, further research is necessary to determine the reasons behind the disparities in the amount of emergency care being purchased in the community and gain a comprehensive understanding of its consequences. While certain variations may be attributed to facility-specific characteristics, it is probable that other factors, such as travel time and distance, patient acuity, and stability, particularly when using ambulance services, also play a role.

### Limitations

To our knowledge, this is the first study to examine trends in the number and types of ED visits occurring in the community vs those provided in the VA. These results should begin to inform the VA about those specific areas where VA acute care remains an important source of care for veterans, and where potential gaps exist. Despite this strength, there are some limitations with this work that should be noted. First, our analysis is based on administrative data. An inherent limitation in the use of administrative data is the possibility of miscoding, variations in coding, and changing definitions. Second, we did not examine veterans’ outcomes and whether receipt of community care, which may involve care across multiple health care systems, has had an effect on care coordination, utilization outcomes, quality of life, and the quality of care.

## Conclusions

In conclusion, our study highlights the swift expansion of the VA’s purchasing of community-based emergency care, a development with significant implications for policy and budget decisions aimed at ensuring veterans’ access to health care. While the VA remains the primary source of emergency care for veterans, it now operates as both a care source and purchaser, necessitating a thorough evaluation of this transformative shift. Future work should focus on assessing its impact, particularly on the quality of health care services delivered in community settings, including patient satisfaction, and health outcomes.

## Notes

Supplement 1.
eMethods.
**eTable 1. **Characteristics of Patients Utilizing Community Emergency Department Visits Purchased by VA, Fiscal Years 2016 to 2022 **eTable 2. **Most Common Conditions Treated in Community Emergency Departments by Fiscal Year, 2016 to 2022 **eTable 3. **Most Costly Conditions Treated in Community Emergency Departments, Fiscal Years 2016 to 2022 **eTable 4. **Proportion of Community Emergency Department Visits by Facility Characteristics, 2016 to 2022 **eTable 5. **Number of Veteran Enrollees by Fiscal Year, 2016 to 2022 **eFigure. **Payments Associated With Community Emergency Department Visits by Fiscal Year

Supplement 2.
Data Sharing Statement
