# Supplementary material for: Community Emergency Care Use by Veterans in an Era of Expanding Choice
Source: JAMA Netw Open. 2024 Mar 8;7(3):e241626. doi: 10.1001/jamanetworkopen.2024.1626 (PMC10924239; doi:10.1001/jamanetworkopen.2024.1626)
Supplement: Supplement 1. — eMethods. eTable 1. Characteristics of Patients Utilizing Community Emergency Department Visits Purchased by VA, Fiscal Years 2016 to 2022 eTable 2. Most Common Conditions Treated in Community Emergency Departments by Fiscal Year, 2016 to 2022 eTable 3. Most Costly Conditions Treated in Community Emergency Departments, Fiscal Years 2016 to 2022 eTable 4. Proportion of Community Emergency Department Visits by Facility Characteristics, 2016 to 2022 eTable 5. Number of Veteran Enrollees by Fiscal Year, 2016 to 2022 eFigure. Payments Associated With Community Emergency Department Visits by Fiscal Year [file jamanetwopen-e241626-s001.pdf]

# Supplemental Online Content

Vashi AA, Urech T, Wu S, Tran LD. Community emergency care use by veterans in an era of expanding choice. *JAMA Netw Open*. 2024;7(3):e241626. doi:10.1001/jamanetworkopen.2024.1626

**eMethods**

**eTable 1.** Characteristics of Patients Utilizing Community Emergency Department Visits Purchased by VA, Fiscal Years 2016 to 2022

**eTable 2.** Most Common Conditions Treated in Community Emergency Departments by Fiscal Year, 2016 to 2022

**eTable 3.** Most Costly Conditions Treated in Community Emergency Departments, Fiscal Years 2016 to 2022

**eTable 4.** Proportion of Community Emergency Department Visits by Facility Characteristics, 2016 to 2022

**eTable 5.** Number of Veteran Enrollees by Fiscal Year, 2016 to 2022

**eFigure.** Payments Associated With Community Emergency Department Visits by Fiscal Year

This supplemental material has been provided by the authors to give readers additional information about their work.

© 2024 Vashi AA et al. *JAMA Network Open.*

## eMethods

The **service-connected disability** rating for Veterans informs both disability compensation as well as benefits eligibility, such as health care and copayment rates; it is based on illnesses or injuries that were sustained or aggravated during military service and is assigned from 0% (least disabling) to 100% (most disabling).

A Veteran was considered **unhoused** during a given FY if the Veteran had any of the following indicated in the CDW: an outpatient visit that included providing services for Veterans at risk of homelessness or currently unhoused, an inpatient stay that was associated with a treating specialty that provided care to unhoused Veterans, or a diagnosis code related to homelessness (e.g., Z59.00, Z59.01).

**Rurality** designation was based on a Veteran's home address and was obtained from the VA Geospatial Service Support Center data.

**Race and ethnicity** were derived from electronic health record (EHR) data from VA national Corporate Data Warehouse (CDW) data transformed into the Observational Medical Outcomes Partnership (OMOP) common data model. Race/ethnicity is acquired through self-report, proxy, and the VHA enrollment coordinator or clerk. The responses are entered into the Computerized Patient Record System (CPRS). OMOP then uses a logic and cleans the data.

The **Facility Complexity Model** uses clinical and administrative data to categorize facilities based on workload and programs (such as teaching, research, and complex clinical programs). Because facility complexities can change over time, we updated a facility's complexity designation as follows: the 2014 facility complexity model designations were used to cover FY 2015-2017, The 2017 facility complexity model designations were used to cover FYs 2018-2020, and the 2020 model designations were used for FYs 2021-2023.

© 2024 Vashi AA et al. *JAMA Network Open.*

**eTable 1. Characteristics of Patients Utilizing Community Emergency Department Visits Purchased by VA, Fiscal Years 2016 to 2022**

| | 2016 | 2017 | 2018 | 2019 | 2020 | 2021 | 2022 | Overall |
|---|---|---|---|---|---|---|---|---|
| | 304512 pts | 335095 pts | 363425 pts | 428312 pts | 525499 pts | 632481 pts | 712111 pts | 1941414 pts |
| Characteristic[a] | | | | | | | | |
| Age, y, mean (sd) | 58.3 (15.5) | 58.6 (15.7) | 59.0 (15.9) | 59.9 (16.2) | 62.0 (16.4) | 62.5 (16.6) | 63.1 (16.8) | 61.4 (16.8) |
| Age group | | | | | | | | |
| 18-35 | 37962 (12.5) | 41595 (12.4) | 43414 (12.0) | 48051 (11.2) | 49513 (9.4) | 56526 (8.9) | 61625 (8.7) | 206277 (10.6) |
| 36-50 | 46053 (15.1) | 51380 (15.3) | 57797 (15.9) | 67715 (15.8) | 76626 (14.6) | 93792 (14.8) | 104614 (14.7) | 283839 (14.6) |
| 51-64 | 97714 (32.1) | 101550 (30.3) | 105134 (28.9) | 116262 (27.1) | 128188 (24.4) | 146424 (23.2) | 157214 (22.1) | 480397 (24.7) |
| 65-75 | 94209 (30.9) | 107375 (32.0) | 118357 (32.6) | 140297 (32.7) | 175576 (33.4) | 208046 (32.9) | 224615 (31.5) | 616784 (31.8) |
| 76-100 | 28465 (9.4) | 33089 (9.9) | 38586 (10.6) | 55790 (13.0) | 95281 (18.1) | 127231 (20.1) | 163460 )23.0) | 352823 (18.2) |
| Sex | | | | | | | | |
| Male | 272459 (89.5) | 299243 (89.3) | 322670 (88.8) | 380924 (88.9) | 472154 (89.9) | 567902 (89.8) | 636053 (89.3) | 1746359 (90.0) |
| Female | 32031 (10.5) | 35839 (10.7) | 40734 (11.2) | 47368 (11.1) | 53324 (10.2) | 64539 (10.2) | 76030 (10.7) | 194943 (10.0) |
| Race | | | | | | | | |
| White | 227542 (74.7) | 248900 (74.3) | 268659 (73.9) | 317548 (74.1) | 397492 (75.6) | 477809 (75.6) | 536186 (75.3) | 1450715 (74.7) |
| Black or AA | 50203 (16.5) | 56633 (16.9) | 62953 (17.3) | 72882 (17.0) | 81810 (15.6) | 97836 (15.5) | 109225 (15.3) | 309532 (15.9) |
| NH or OPI | 2944 (1.0) | 3491 (1.0) | 3689 (1.0) | 4264 (1.0) | 4698 (0.9) | 5572 (0.9) | 6088 (0.9) | 17407 (0.9) |
| AI or AN | 3185 (1.1) | 3614 (1.1) | 3781 (1.0) | 4504 (1.1) | 5369 (1.0) | 6158 (1.0) | 6915 (1.0) | 18886 (1.0) |
| Asian | 1653 (0.5) | 2032 (0.6) | 2143 (0.6) | 2648 (0.6) | 2951 (0.6) | 3493 (0.6) | 4285 (0.6) | 12705 (0.7) |
| Unknown | 18985 (6.2) | 20425 (6.1) | 22200 (6.1) | 26466 (6.2) | 33179 (6.3) | 41613 (6.6) | 49412 (6.9) | 132169 (6.8) |
| Ethnicity | | | | | | | | |
| Hispanic or Latino | 17953 (5.9) | 20158 (6.0) | 22361 (6.2) | 26601 (6.2) | 29039 (5.5) | 35028 (5.5) | 38722 (5.4) | 111449 (5.7) |
| Not Hispanic or Latino | 279107 (91.7) | 306706 (91.5) | 332026 (91.4) | 390418 (91.2) | 480826 (91.5) | 572782 (90.6) | 630684 (88.6) | 1745208 (89.9) |
| Unknown | 7452 (2.5) | 8231 (2.5) | 9038 (2.5) | 11293 (2.6) | 15634 (2.9) | 24671 (3.9) | 42705 (6.0) | 84757 (4.4) |
| Unhoused | 32401 (10.6) | 34351 (10.3) | 35241(9.7) | 37420 (8.7) | 39047 (7.4) | 41659 (6.6) | 43997 (6.2) | 138201 (7.1) |
| Rurality | | | | | | | | |
| Urban | 180110 (59.2) | 197062 (58.8) | 213214 (58.7) | 248425 (58.0) | 295559 (56.2) | 354261 (56.0) | 391520 (55.0) | 1119649 (57.7) |
| Rural | 123633 (40.6) | 136629 (40.8) | 149133 (41.0) | 178560 (41.7) | 228736 (43.5) | 277071 (43.8) | 311723 (43.8) | 808919 (41.7) |
| Insular | 258 (0.1) | 407 (0.1) | 457 (0.1) | 595 (0.1) | 523 (0.1) | 530 (0.1) | 604 (0.1) | 1822 (0.1) |
| VA priority group[b] | | | | | | | | |
| Highly disabled | 157002 (51.6) | 177789 (53.1) | 201144 (55.4) | 235774 (55.1) | 268483 (51.1) | 322792 (51.0) | 377915 (53.1) | 963746 (49.6) |
| Low/moderate disability | 50314 (16.5) | 54418 (16.2) | 57585 (15.9) | 69731 (16.3) | 93408 (17.8) | 115330 (18.2) | 129232 (18.2) | 360227 (18.6) |
| Low income | 74405 (24.4) | 77241 (23.1) | 77710 21.38 | 88779 (20.7) | 110368 (21.0) | 123347 (19.5) | 126687 (17.8) | 405267 (20.9) |
| Non-disabled | 22566 (7.4) | 25386 (7.6) | 26755 (7.4) | 33654 (7.9) | 52827 (10.1) | 70634 (11.17) | 78016 (11.0) | 210518 (10.8) |
| VA SC disability rating | | | | | | | | |

© 2024 Vashi AA et al. *JAMA Network Open.*

| | | | | | | | | |
|---|---|---|---|---|---|---|---|---|
| No SC disability | 106860 (35.1) | 112445 (33.6) | 114878 (31.6) | 134320 (31.4) | 181524 (34.5) | 215971 (34.2) | 228277 (32.1) | 683268 (35.2) |
| SC 0-49% | 52513 (17.2) | 56829 (17.0) | 59853 (16.5) | 72199 (16.9) | 94477 (18.0) | 115085 (18.2) | 128177 (18.0) | 358669 (18.5) |
| SC 50-99% | 89121 (29.3) | 101017 (30.2) | 113119 (31.1) | 132394 (30.9) | 151610 (28.9) | 182168 (28.8) | 208728 (29.3) | 553626 (28.5) |
| SC 100% | 55317 (18.2) | 64004 (19.1) | 74628 (20.5) | 88003 (20.6) | 95768 (18.2) | 116621 (18.4) | 143345 (20.1) | 337726 (17.4) |
| Elixhauser comorbidity score, mean (sd) | 3.9 (2.6) | 4.0 (28) | 4.2 (3.0) | 4.3 (3.1) | 4.0 (3.1) | 3.9 (3.0) | 3.6 (2.6) | 3.8 (2.8) |
| Elixhauser conditions | | | | | | | | |
| 0-1 | 80873 (26.6) | 89440 (26.7) | 93564 (25.7) | 108905 (25.4) | 155910 (29.7) | 195729 (31.0) | 238476 (33.5) | 584214 (30.1) |
| 2-3 | 97932 (32.2) | 105735 (31.6) | 110847 (30.5) | 130316 (30.4) | 164109 (31.2) | 200824 (31.8) | 233065 (32.7) | 633043 (32.6) |
| 4-6 | 85431 (28.1) | 92313 (27.6) | 101214 (27.9) | 118840 (27.8) | 132556 (25.2) | 155149 (24.5) | 171390 (24.1) | 479948 (24.7) |
| 7+ | 40276 (13.2) | 47607 (14.2) | 57800 (15.9) | 70251 (16.4) | 72924 (13.9) | 80779 (12.8) | 69180 (9.7) | 244209 (12.6) |

Abbreviations: AA, African American; AI, American Indian; AN, Alaska Native; ED, emergency department, NH, Native Hawaiian; OPI, Other Pacific Islander; SC, service-connected; VA, Veterans Affairs.

[a]Values expressed as counts (percentages) unless otherwise indicated. Missing values for age, sex, rurality, VA priority group, and VA SC disability characteristics were less than 1.0% and are not reported in the table.

[b]Highly disabled defined as VA Priority Groups 1 and 4. Low/moderate disability defined as VA Priority Groups 2, 3, and 6. Low income defined as VA Priority Group 5. Non-disabled defined as VA Priority Groups 7 and 8.

## eTable 2. Most Common Conditions Treated in Community Emergency Departments by Fiscal Year, 2016 to 2022

| | **Number of ED Visits by Fiscal Year** | | | | | | |
|---|---|---|---|---|---|---|---|
| | **2016** | **2017** | **2018** | **2019** | **2020** | **2021** | **2022** |
| **Rank** | **465,253** | **522,280** | **565,014** | **674,639** | **828,362** | **1,019,141** | **1,180,106** |
| 1 | Nonspecific chest pain (7.3)[a] | Nonspecific chest pain (6.7) | Nonspecific chest pain (6.5) | Nonspecific chest pain (6.2) | Nonspecific chest pain (5.6) | Nonspecific chest pain (5.2) | Nonspecific chest pain (5.0) |
| 2 | Abdominal pain (3.7) | Abdominal pain (3.5) | Abdominal pain (3.4) | Abdominal pain (3.4) | Abdominal pain (3.3) | COVID-19 (4.5) | COVID-19 (4.7) |
| 3 | COPD[b] (2.7) | COPD (3.0) | COPD (2.7) | Musculoskeletal pain, not low back pain (2.6) | Septicemia (3.2) | Septicemia (3.3) | Abdominal pain (3.2) |
| 4 | Superficial injury; contusion (2.4) | Superficial injury; contusion (2.5) | Superficial injury; contusion (2.4) | COPD (2.6) | Musculoskeletal pain, not low back pain (2.5) | Abdominal pain (3.2) | Septicemia (3.2) |
| 5 | Musculoskeletal pain, not low back pain (2.2) | Septicemia (2.3) | Septicemia (2.4) | Septicemia (2.5) | Heart failure (2.4) | Musculoskeletal pain, not low back pain (2.7) | Musculoskeletal pain, not low back pain (2.9) |
| 6 | Sprains and strains (2.2) | Musculoskeletal pain, not low back pain (2.3) | Musculoskeletal pain, not low back pain (2.3) | Superficial injury; contusion (2.5) | COPD (2.4) | Heart failure (2.3) | Superficial injury; contusion (2.4) |

© 2024 Vashi AA et al. *JAMA Network Open.*

| | | | | | | | |
|---|---|---|---|---|---|---|---|
| 7 | Skin and subcutaneous tissue infections (2.2) | Skin and subcutaneous tissue infections (2.2) | Skin and subcutaneous tissue infections (2.2) | Skin and subcutaneous tissue infections (2.3) | Respiratory signs and symptoms (2.3) | Superficial injury; contusion (2.3) | Heart failure (2.3) |
| 8 | Septicemia (2.2) | Sprains and strains (2.2) | Sprains and strains (2.1) | Sprains and strains (2.2) | Superficial injury; contusion (2.3) | Respiratory signs and symptoms (2.1) | Respiratory signs and symptoms (2.0) |
| 9 | Open wounds to limbs (2.0) | Heart failure (2.1) | Heart failure (2.1) | Heart failure (2.1) | Skin and subcutaneous tissue infections (2.1) | Skin and subcutaneous tissue infections (2.1) | Skin and subcutaneous tissue infections (2.0) |
| 10 | Alcohol-related disorders (2.0) | Alcohol-related disorders (2.0) | Open wounds to limbs (2.0) | Respiratory signs and symptoms (2.0) | Sprains and strains (1.9) | Sprains and strains (1.9) | Urinary tract infections (2.0) |

Abbreviation: ED, emergency department.

[a]Condition groups are based on the Clinical Classification Software Refined (CCSR) tool. Numbers indicate percentages.

[b]Includes chronic obstructive pulmonary disease (COPD) and bronchiectasis.

© 2024 Vashi AA et al. *JAMA Network Open.*

## eTable 3. Most Costly Conditions Treated in Community Emergency Departments, Fiscal Years 2016 to 2022

| **Community ED Payments (n=$21.3 billion)** | | |
|---|---|---|
| **Rank** | **CCSR Condition Category** | **%** |
| 1 | Septicemia | 12.7 |
| 2 | Acute myocardial infarction | 4.8 |
| 3 | Coronavirus disease – 2019 (COVID-19) | 4.6 |
| 4 | Heart failure | 4.6 |
| 5 | Cerebral infarction | 2.9 |
| 6 | Cardiac dysrhythmias | 2.3 |
| 7 | Respiratory failure; insufficiency; arrest | 2.3 |
| 8 | Diabetes mellitus with complication | 2.2 |
| 9 | Nonspecific chest pain | 2.1 |
| 10 | Pneumonia | 2.0 |

Abbreviation: CCSR, Clinical Classification Software Refined; ED, emergency department

© 2024 Vashi AA et al. *JAMA Network Open.*

**eTable 4. Proportion of Community Emergency Department Visits by Facility Characteristics, 2016 to 2022**

| | n | Median | Interquartile Range |
|---|---|---|---|
| Fiscal Year ($\chi 2$: 227.1; p-value<.001) | | | |
| 2016 | 107 | 12.7% | 9.4% |
| 2017 | 106 | 13.5% | 9.7% |
| 2018 | 108 | 15.4% | 10.1% |
| 2019 | 108 | 17.0% | 11.5% |
| 2020 | 107 | 22.5% | 17.0% |
| 2021 | 107 | 27.0% | 20.0% |
| 2022 | 107 | 30.2% | 20.1% |
| EC Visit Volume ($\chi 2$: 29.5; p-value<.001) | | | |
| <=10,000 | 29 | 19.3% | 8.9% |
| 10,001-20,000 | 267 | 20.8% | 18.6% |
| 20,001-30,000 | 241 | 16.2% | 11.7% |
| 30,001+ | 213 | 23.2% | 16.8% |
| Facility Complexity ($\chi 2$: 71.6; p-value<.001) | | | |
| 1a-High Complexity | 273 | 19.5% | 14.5% |
| 1b-High Complexity | 145 | 16.2% | 10.9% |
| 1c-High Complexity | 182 | 16.5% | 17.6% |
| 2-Medium Complexity | 127 | 25.4% | 20.5% |
| 3-Low Complexity | 23 | 34.5% | 16.5% |
| Teaching Status (z-score: 5.7; p-value<.001) | | | |
| Non-Teaching | 350 | 21.8% | 18.4% |
| Teaching Status | 400 | 17.2% | 14.3% |
| Rural Status (z-score: -5.1; p-value<.001) | | | |
| Urban | 689 | 18.3% | 14.9% |
| Rural | 61 | 27.5% | 21.9% |
| U.S. Census Division ($\chi 2$: 84.4; p-value<.001) | | | |
| East North Central | 89 | 14.5% | 14.8% |
| East South Central | 64 | 26.1% | 24.3% |
| Middle Atlantic | 77 | 11.7% | 11.7% |
| Mountain | 84 | 20.4% | 15.9% |
| New England | 35 | 22.2% | 13.0% |
| Pacific | 74 | 20.0% | 14.1% |
| South Atlantic | 168 | 18.1% | 15.6% |
| West North Central | 84 | 20.2% | 11.7% |
| West South Central | 75 | 24.1% | 16.2% |

© 2024 Vashi AA et al. *JAMA Network Open.*

**eTable 5. Number of Veteran Enrollees by Fiscal Year, 2016 to 2022**

| Fiscal Year | N |
|---|---|
| 2016 | 9,752,530 |
| 2017 | 9,847,244 |
| 2018 | 9,898,766 |
| 2019 | 9,930,287 |
| 2020 | 9,888,915 |
| 2021 | 10,010,358 |
| 2022 | 9,798,147 |

© 2024 Vashi AA et al. *JAMA Network Open.*

**eFigure. Payments Associated With Community Emergency Department Visits by Fiscal Year**

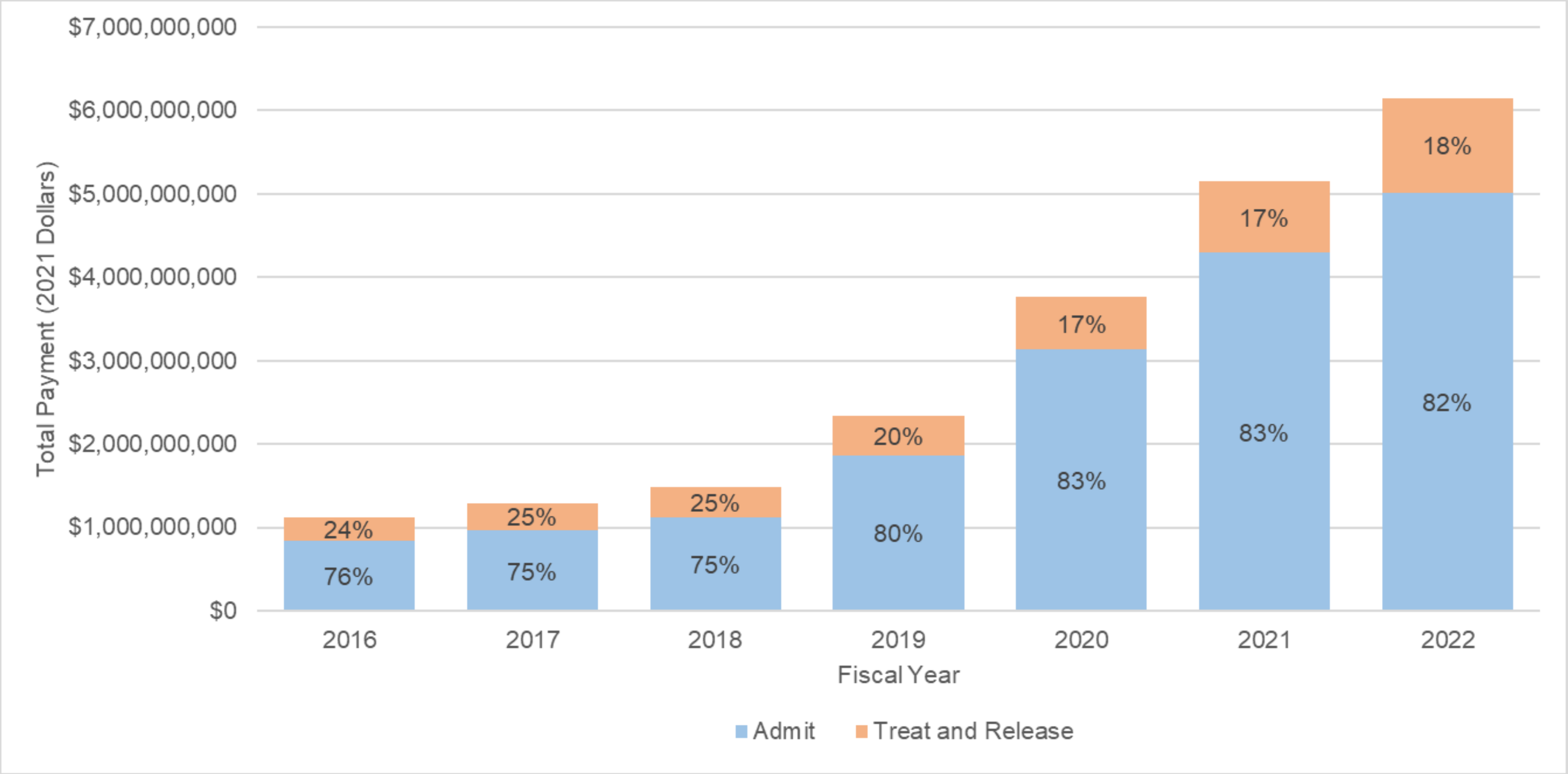


Abbreviation: ED, emergency department.
Note: Payments for ED visits that resulted in death within the same day comprised 0.1 to 0.2% of the total payments across the years examined.

© 2024 Vashi AA et al. *JAMA Network Open.*
